# Detection of SFTS virus RNA and antibodies in severe fever with thrombocytopenia syndrome surveillance cases in endemic areas of China

**DOI:** 10.1186/s12879-019-4068-2

**Published:** 2019-05-28

**Authors:** Xiaoxia Huang, Shujun Ding, Xiaolin Jiang, Bo Pang, Quanfu Zhang, Chuan Li, Aqian Li, Jiandong Li, Mifang Liang, Shiwen Wang, Dexin Li

**Affiliations:** 1 NHC Key Laboratory of Medical Viruses and Viral Diseases, National Institute for Viral Disease Control and Prevention, China CDC, Beijing, China; 2 Shandong Provincial Key Laboratory of Communicable Disease Control and Prevention, Shandong Center for Disease Control and Prevention, Shandong Province, Jinan, China

**Keywords:** SFTS, SFTSV antibodies, Surveillance cases

## Abstract

**Background:**

Severe fever with thrombocytopenia syndrome (SFTS) is a newly identified severe infectious disease caused by SFTS phlebovirus (SFTSV). SFTS monitoring has been carried out since 2010 in mainland China. We analysed the detection results of SFTSV RNA and antibody in SFTS surveillance cases to provide basic data for SFTS diagnosis.

**Methods:**

This study was conducted in Shandong Province. Sera of SFTS surveillance cases were collected to detect SFTSV RNA and antibody by real-time RT-PCR and enzyme-linked immunosorbent assay, respectively. Detection rates were calculated. SPSS 18.0 (Chicago, IL, USA) was used for statistical analysis to compare the detection rates of SFTSV RNA and antibodies among different sera groups.

**Results:**

A total of 374 SFTS surveillance cases were enrolled. Overall, 93.3% (349/374) of the sera samples were collected within 2 weeks after onset, and 6.7% (25/374) were collected between 15 days and 45 days. Of these, 183 (48.9%) were positive for SFTSV RNA. The SFTSV RNA-positive rate peaked (52.2%) in samples collected ≤7 days after onset and then showed a decreasing trend. The detection rate of SFTSV-specific IgM antibody was 30.5% (46/151) and was highest in samples collected between 8 and 14 days (43.3%, 26/60). The positive rate of SFTSV-specific IgG antibody (17.9%, 27/151) showed an increasing trend with the specimen collection time. In total, 74.8% (113/151) of sera samples had the same SFTSV RNA and IgM antibody detection results. However, 23.2% (29/125) of SFTSV RNA-negative cases were IgM antibody-positive, and 8.6% (9/105) of IgM antibody-negative cases were SFTSV RNA-positive.

**Conclusions:**

SFTSV RNA detection was preferred for SFTSV infection during disease surveillance. For highly suspected SFTS cases, IgM antibody is suggested to make a comprehensive judgement.

## Background

Severe fever with thrombocytopenia syndrome (SFTS), which is mainly characterised by fever, thrombocytopenia, and leukocytopenia, is an infectious disease first identified in China in 2009 [1]. Confirmed cases have also been reported in other Asian countries (Japan and South Korea) [2, 3]. In China, most of the SFTS cases are farmers aged 40–79 years in seven provinces of central and eastern China [1, 4]. The average fatality rate is nearly 8%, but it varies in different populations, reaching 30% [5]. Although SFTS is a tick-borne disease, person-to-person transmission caused by direct contact with blood has also been reported [6–8]. It is still a severe threat to public health.

SFTS phlebovirus (SFTSV) in the *Phlebovirus* genus of the *Phenuiviridae* family has been identified as the causative agent. Virus RNA detection by real-time RT-PCR and antibody detection by enzyme-linked immunosorbent assay (ELISA) are commonly used to identify virus infection. The former is often used to confirm SFTSV infection. However, a previous study [9] in Henan Province showed an approximately 50% positive rate of SFTSV RNA in SFTS surveillance cases, and 14% of cases with SFTSV-specific IgM antibodies were observed in a group of RNA-negative cases. More information is necessary about the detection of SFTSV RNA and antibodies (especially IgM antibody) in the early stage after disease onset.

Shandong Province is a high epidemic area, with 1074 reported SFTS cases between 2011 and 2014, of which nearly 30% did not have laboratory evidence [4]. The detection results of SFTSV RNA or antibodies in routine SFTS monitoring were not very clear. To fill this gap, we performed SFTSV RNA and antibody detection and analysis on the acute phase sera of SFTS surveillance cases collected in Shandong Province in 2014. The aim was to understand the detection results of SFTSV RNA and antibodies and to explore appropriate conventional laboratory pathogenic detection strategies to provide a pathogenic and serological basis for better diagnosis of SFTS cases.

## Methods

### Sample collection

A total of 374 sera samples were collected from SFTS surveillance cases distributed in 14 cities of Shandong Province in 2014. Here, SFTS surveillance cases were suspected SFTS cases or clinically diagnosed SFTS cases that required further laboratory detection. General information (e.g., gender, age, occupation, and residence type), epidemiological information (e.g., tick bite history) and clinical manifestation (e.g., body temperature, platelet count, leukocyte count, and lymphadenopathy) from each case were extracted from a well-written questionnaire. Specimens were divided into three groups according to sampling days after onset: ≤7 days (Group A), 8–14 days (Group B), and ≥ 15 days (Group C); Group AB (≤14 days) represents Group A plus Group B.

### SFTSV RNA detection

A total of 140 μl of serum was used for RNA extraction with the RNeasy Mini Kit (Qiagen, Germany). Real-time RT-PCR was conducted with the SuperScript III Platinum One-Step Quantitative RT-PCR System Kit (Invitrogen, USA). We followed the kit instructions to conduct the experiments. Reaction parameters were 50 °C for 30 min, 95 °C for 2 min, and then 40 cycles of 95 °C for 15 s and 60 °C for 30 s. The primers and probes located in the L, M, and S segments of SFTSV were from a previous study [10]. The cut-off cycle threshold (Ct) value was set at 35 cycles.

### SFTSV antibody detection

Sera were tested for SFTSV-specific IgM and IgG antibodies using an ELISA kit (Zhongshan Bio-Tech Co., Ltd.) based on the procedures described previously [11]. In brief, 100 μl of diluted serum, 100 μl of horseradish peroxidase (HRP)-labelled enzyme conjugate, and the chromogenic substrate were added to each well. Finally, the optical density (OD) value was read at 450 nm with a microplate reader (Thermo). The cut-off value was set at the average OD value of the negative controls plus 0.10.

### Statistical analysis

SPSS 18.0 (Chicago, IL, USA) was used for statistical analysis. Pearson chi-square test was conducted to compare the detection rates of SFTSV RNA and antibodies among samples collected on different days. The McNemar test was used to compare SFTSV RNA and IgM antibody detection rates in the same samples, and the kappa value was calculated to compare the detection consistency of the two methods.

### Ethical statement

This study was approved by the ethics committee of the National Institute for Viral Disease Control and Prevention, Chinese Center for Disease Control and Prevention. Written informed consent was obtained from the study participants.

## Results

### Characteristics of SFTS surveillance cases

A total of 374 SFTS surveillance cases were enrolled in this study, mainly distributed in Yantai (156), Weihai (78), Weifang (35), Jinan (28), and Zibo (22) of Shandong Province. The median age of participants was 59 years (range, 7–88 years); 52.8% (197/373) were male; of the 289 cases with occupation information, the top three occupations were farmers (81.3%), workers (5.9%), and household workers and unemployed (3.8%). Ninety-nine percent had a fever. A total of 93.3% (349/374) of the samples were collected within 2 weeks after onset, and the remaining 6.7% (25/374) were collected between 15 days and 45 days. The median and interquartile range (25%, 75%) of the collection days after onset of the 374 samples was 7 days (4–9 days). The major clinical and epidemiological information of the participants is shown in Table 1.

**Table 1:** Major clinical and epidemiological information of participants

Variable	Normal level	Observed no.	No. (%)	Median and interquartile range (25%, 75%)
Fever (> 37.3 °C)	–	302	299 (99)	NA
With gastrointestinal symptoms	–	288	253 (87.8)	NA
Lymphadenopathy	–	298	56 (18.8)	NA
With tick bite history	–	299	36 (12)	NA
With fatigue	–	302	254 (84.1)	NA
Leukocyte count, 10^9^/L	4–10	257	NA	2.7 (1.9–4.6)
Platelet count, 10^9^/L	100–300	254	NA	69 (47–92)

### SFTSV RNA detection

Of the 374 samples, 183 (48.9%) were positive for SFTSV RNA (Table 2). SFTSV RNA was detected on the day of onset and until the 27th day after onset. The positive rate of SFTSV RNA among Group A was 52.2%, which decreased with the increase in the time interval between specimen collections. The positive rate of SFTSV RNA in Group C was 28%. There was no statistically significant difference (χ2 = 0.818, *P* = 0.366) between Group A and Group B. However, a significant difference was found in specimens collected between Group AB and Group C (χ2 = 4.697, *P* = 0.03).

**Table 2:** SFTSV RNA, IgM and IgG antibody detection results

Sampling days	SFTSV RNA-positive samples no. / total detection no. (%)	SFTSV IgM antibody-positive samples no. / total detection no. (%)	SFTSV IgG antibody-positive samples no. / total detection no. (%)
≤7 days	119/228(52.2)	15/76(19.7)	11/76(14.5)
8–14 days	57/121(47.1)	26/60(43.3)	11/60(18.3)
≥15 days	7/25(28)	5/15(33.3)	5/15(33.3)
Total	183/374(48.9)	46/151(30.5)	27/151(17.9)

### SFTSV antibody detection

Of the 374 cases, 151 sera samples were chosen to conduct SFTSV antibody (IgM and IgG) detection. The median collection time of these specimens was 7 days (range 0–45 days). The detection rate of SFTSV-specific IgM antibody was 30.5% (46/151). IgM antibodies were detected on the onset day and could still be detected 45 days after onset. The highest IgM antibody detection rate was found in Group B (43.3%, 26/60). There was a difference in the detection rate of IgM between Group A and Group B (χ2 = 8.865, *P* = 0.003). The positive rate of SFTSV-specific IgG antibody was 17.9% (27/151). The detection rate of IgG antibody showed an increasing trend with the sampling days. There was no statistically significant difference in the detection rate of IgG antibodies among Groups A, B, and C (χ2 = 3.048, *P* = 0.218). Table 2 show the antibody detection results.

### Comparison of SFTSV RNA and IgM antibody detection

The detection rates of SFTSV IgM antibodies were 65.4% (17/26) and 23.2% (29/125) in SFTSV RNA-positive and SFTSV RNA-negative samples, respectively (Table 3). A total of 74.8% (113/151) of the samples had the same SFTSV RNA and IgM antibody detection results. However, there was a significant difference between the detection rates of SFTSV RNA and IgM antibody, and the detection consistency was poor.

**Table 3:** Comparison of SFTSV RNA and IgM antibody detection

Days after onset	SFTSV RNA	SFTSV IgM		*P* value (McNemar test)	Kappa value
		Positive	Negative		
		N (%)	N (%)		
≤14 days	Positive	15(62.5)	9(37.5)	0.06	0.307
	Negative	26 (23.2)	86(76.8)		
≥15 days	Positive	2(100)	0(0)	0.25	0.471
	Negative	3(23.1)	10(76.9)		
Total	Positive	17(65.4)	9(34.6)	0.002	0.323
	Negative	29(23.2)	96(76.8)		

## Discussion

Pathogen detection mainly targets RNA and antibodies. Real-time RT-PCR is a commonly used method for virus RNA detection, which directly targets the RNA of pathogens. ELISA is often used for antibody detection, which is an indirect method to determine whether virus infection exists by detecting virus antibodies (IgM and IgG). The former has a higher detection sensitivity for specimens collected in the acute phase [10]; however, it has higher requirements for the testing environment and skilled personnel. ELISA requires a simpler operating environment and is much easier to operate by professionals at the county level. These two methods are often used in the early diagnosis of pathogens. Therefore, we used these two methods to detect SFTSV RNA and antibodies (IgM and IgG) in this study.

Shandong Province is a highly endemic area for SFTS and where SFTSV RNA detection is mainly used to confirm SFTSV infection during surveillance. Here, we compared the detection rates of SFTSV RNA and SFTSV-specific antibodies. Approximately 49% of SFTS surveillance cases had SFTSV RNA, and 30.5% had SFTSV-specific IgM antibodies, which was similar to other recorded data [9]. SFTSV RNA was detected on the onset day, and the detection rate, which was highest within the first week (52.2%), decreased with sampling time. The detection rate of SFTSV IgM antibody was higher in the second week. The SFTSV RNA detection rate was much higher than that of IgM antibody for samples collected within two weeks after onset. Therefore, SFTSV RNA should be given priority in the early detection of SFTSV. For SFTSV RNA detection, serum collection during the acute phase (within two weeks after disease onset) of disease was often recommended; therefore, we compared the SFTSV RNA detection and IgM antibody detection results in two groups (≤14 days and ≥ 15 days). This study showed that the detection rates between SFTSV RNA and IgM were different, and the detection consistency was poor. Nearly a quarter of the cases negative for SFTSV RNA were positive for SFTSV IgM antibody, which was not observed in healthy people [12]. These results implied that for SFTSV RNA-negative cases, exclusion should be carefully considered. In addition, 8.6% of SFTSV IgM antibody-negative cases were positive for SFTSV RNA. Therefore, SFTSV IgM antibody-negative cases could not be completely ruled out as SFTS when only relying on IgM antibody detection. The positive rate of SFTSV IgG antibody was only 17.9%, and it maintained at a relatively low level within one and a half months after disease onset. Paired specimens were required for SFTSV infection confirmed with IgG antibody, which was difficult to collect; therefore, IgG antibody testing was generally not used for early routine case monitoring.

SFTSV RNA detection was superior to IgM antibody detection; however, undetected phenomenon in real SFTS cases may exist both in SFTSV detection alone and in SFTSV IgM detection alone. It has been reported that the IgM antibody-positive rate was very high in confirmed SFTS cases [9, 13]. However, because IgM antibodies exist for a long time after onset [14], SFTSV IgM antibody positivity alone might not be sufficient evidence for current infection. Therefore, it is suggested that SFTSV RNA detection is preferred for SFTSV infection during disease surveillance. For highly suspected SFTS cases, testing for IgM antibody is suggested to make a comprehensive judgement.

To interpret the results of the study, the following limitations should be considered: first, for the detection rate analysis, three groups (0–7 days, 8–14 days and ≥ 15 days) were divided according to sampling days. The ≥15 days group had a relatively small number of cases, and the time interval between specimen collection and onset was relatively wide (15–45 days), which might cause bias in the statistical analysis. Second, considering that there might be missed SFTS cases in the SFTSV RNA-negative cases, more SFTSV RNA-negative cases were chosen to conduct antibody detection, which might lead to a certain bias in the detection rate comparison between these two methods.

## Conclusions

By analysing the detection rate of SFTSV RNA and antibodies conducted with real-time RT-PCR and ELISA, our study suggested that SFTSV RNA testing was the preferred method during SFTS surveillance. However, for highly suspected cases that are SFTSV RNA-negative, case exclusion should be carefully considered, and IgM antibody detection is suggested to provide further information for case exclusion.

## Abbreviations

ELISA: Enzyme-linked immunosorbent assay
SFTS: Severe fever with thrombocytopenia syndrome
SFTSV: SFTS phlebovirus
